# Electroacupuncture Exerts Neuroprotection through Caveolin-1 Mediated Molecular Pathway in Intracerebral Hemorrhage of Rats

**DOI:** 10.1155/2016/7308261

**Published:** 2016-09-20

**Authors:** Hui-Qin Li, Yan Li, Zi-Xian Chen, Xiao-Guang Zhang, Xia-wei Zheng, Wen-ting Yang, Shuang Chen, Guo-Qing Zheng

**Affiliations:** Department of Neurology, The Second Affiliated Hospital and Yuying Children's Hospital of Wenzhou Medical University, Wenzhou, China

## Abstract

Spontaneous intracerebral hemorrhage (ICH) is one of the most devastating types of stroke. Here, we aim to demonstrate that electroacupuncture on Baihui (GV20) exerts neuroprotection for acute ICH possibly via the caveolin-1/matrix metalloproteinase/blood-brain barrier permeability pathway. The model of ICH was established by using collagenase VII. Rats were randomly divided into three groups: Sham-operation group, Sham electroacupuncture group, and electroacupuncture group. Each group was further divided into 4 subgroups according to the time points of 6 h, 1 d, 3 d, and 7 d after ICH. The methods were used including examination of neurological deficit scores according to Longa's scale, measurement of blood-brain barrier permeability through Evans Blue content,* in situ *immunofluorescent detection of caveolin-1 in brains, western blot analysis of caveolin-1 in brains, and* in situ *zymography for measuring matrix metalloproteinase-2/9 activity in brains. Compared with Sham electroacupuncture group, electroacupuncture group has resulted in a significant improvement in neurological deficit scores and in a reduction in Evans Blue content, expression of caveolin-1, and activity of matrix metalloproteinase-2/9 at 6 h, 1 d, 3 d, and 7 d after ICH (*P* < 0.05). In conclusion, the present results suggested that electroacupuncture on GV20 can improve neurological deficit scores and reduce blood-brain barrier permeability after ICH, and the mechanism possibly targets caveolin-1/matrix metalloproteinase/blood-brain barrier permeability pathway.

## 1. Introduction

Intracerebral hemorrhage (ICH) is one of the leading causes of human death with high morbidity, fatality, and disability, which accounts for 10%~15% of all strokes worldwide [1]. The overall incidence of ICH was 24.6 per 100 000 person-years and the median case fatality at 1 month was 40.4% [2]. Even surviving the ictus, most patients' neurological deficits remain and no more than 40% patients are independent at 6 months [2]. Over the past twenty years, more and more animal and clinical studies have been done to identify the mechanism underlying ICH-induced brain injury, which is considered to be composed of primary injury and secondary injury [3]. According to the primary injury, we should remove the clot or prevent the expansion of haematoma to reduce the physical effects of the haematoma. However, the usefulness of clot evacuation is uncertain for most ICH patients, and there is high thromboembolic risk with hemostatic agents such as recombinant activated factor VII and no clear clinical benefit to ICH patients without coagulopathy [4]. In addition, although there are a cluster of potential therapeutic targets for preventing ICH secondary brain injury, the relevant recommendations are merely symptomatic and supportive [4]. Therefore, more and more patients resort to complementary and alternative medicines (CAM) for ICH.

Acupuncture, as one form of CAM, has a long history worldwide [5] and its efficacy for treating stroke is acknowledged [6]. Scalp acupuncture (SA) is a new branch of acupuncture that developed according to traditional acupuncture science in combination with modern anatomy, neurophysiology, and bioholographic theory [7]. It belongs to micropuncture system, in which filiform needle is utilized to penetrate specific stimulation areas of the scalp [8]. Historically, SA has been used to treat various diseases for thousands of years through needling and stimulating the specific areas of the scalp, but SA develops so fast in recent decades. In 1983, Western Pacific Ocean Region Committee of World Health Organization (WHO) entrusted China Acupuncture Association to prepare the scheme of Standard Nomenclature of SA lines. In 1984, 1985, and 1987, after the discussion in the standardization working group, consensus of opinion had been reached and named as “A Proposed Standard International Acupuncture Nomenclature: 3.6 Scalp Acupuncture Lines.” In 1989, this scheme was formally adopted in a science group meeting held by WHO. In 1991, the formal version of SA lines was published [9]. A meta-analysis in our group has showed that SA probably can improve neurological deficits in acute ICH patients [10]. In addition, the GV20 is supposed to be the most important acupuncture point for acute ICH in the rat models [11]. However, the underlying mechanism of SA for acute ICH is not completely clear.

Blood-brain barrier (BBB) plays a key role in the ICH secondary brain injury. A range of factors such as thrombin, chemokines, and matrix metalloproteinases (MMPs) have been implicated in induction of BBB disruption [12–14]. Therefore, preventing BBB disruption like blocking multiple pathways or blocking the common end pathway is a main method to prevent ICH damage. Caveolin-1 (Cav-1) is the main structural protein of caveolae in the cell plasma membrane [15]. It is particularly abundant in endothelial cells, fibroblasts, epithelial cells, and smooth muscle cells [16]. Cav-1 has many important functions such as regulating various signaling molecules, participating in cellular cholesterol transport, and maintaining homeostasis [17]. Cav-1 is also considered as regulation of expression of tight junction-associated proteins in brain microvascular endothelial cells [18]. What is more, Cav-1 has been reported to play an important role in regulating BBB permeability in experimental cerebral ischemia/reperfusion injury [19, 20]. These evidences suggest that Cav-1 could play an important role in brain damage after stroke.

MMPs are a cluster of proteolytic zinc-containing enzymes that can degrade the extracellular matrix around cerebral blood vessels and neurons [21]. Furthermore, MMPs could increase BBB permeability by degrading tight junction proteins [22]. It is considered that activation of MMPs is a key step in the BBB opening [22]. Administration of MMPs inhibitor, BB-1101, could significantly reduce the brain water and sodium content in the ICH models [23]. Our previous study showed that MMPs activity could be regulated by Cav-1 and the BBB permeability could be regulated via Cav-1/MMPs pathway [19]. Thus, in the present study, we hypothesized that electroacupuncture (EA) on Baihui (GV20) exerts neuroprotection for acute ICH possibly via the Cav-1/MMP/BBB permeability pathway.

## 2. Materials and Methods

### 2.1. Ethics Statement

All animal experiments were conducted in accordance with the Guide for the Care and Use of Laboratory Animals issued by the US National Institutes of Health (publication number 85-23). All of animal experimental protocols were approved and regulated by the local ethic committee of the Wenzhou Medical University on the use of live animals in teaching and research (number wydw2014-0104). All the animals were sacrificed by anesthesia at the end of the experiment.

### 2.2. Animals, Grouping, and Induction of ICH Model

Male adult Sprague-Dawley (SD) rats were obtained from Shanghai Laboratory Animal Center (number, SCXK, Shanghai, 2013-148). Rats were randomly divided into three groups: Sham-operation group, Sham electroacupuncture (EA) group, and EA group. Each group was further divided into 4 subgroups according to the time points of 6 h, 1 d, 3 d, and 7 d after ICH. The model of ICH was established as previously described by using collagenase VII [24]. Briefly, SD rats were anaesthetized with 10% chloral hydrate intraperitoneal injection (400 mg/kg). After shaving and regular sterilization, the rats were placed in the stereotactic frame. The scalp was incised longitudinally with 0.8 cm long incision in the midline, and a 0.8 mm burr hole was made in the skull using a dental drill at the point that was 0.2 mm posterior and 2.9 mm lateral to bregma. A sterile needle of 0.7 mm in diameter was then punctured down into a point that was 6 mm ventral to the right caudate nucleus. Normal saline 3 *μ*L containing collagenase VII 0.6 U/*μ*L was injected into right caudate nucleus of rat in Sham EA group and EA group. Then the needle was removed and the scalp sutured together. All rats were allowed free access to food and water. For the Sham-operation group, the same procedure was carried out in the rats without injection of collagenase VII.

### 2.3. EA Treatment

The EA treatment was conducted according to the methodological standards published in our group previously [25]. After the animal was fastened to a frame, a sterile acupuncture needle with a diameter of 0.25 mm was inserted into GV20 towards Qubin (GB7), and another needle was inserted into wet gauze fastened to the tail. Then both of the needles were connected to the type G-6805 EA stimulator. The needles were stimulated with intensity of 0.2 mA, frequency of 2 Hz, and stimulation duration of 30 min. EA treatment in each group was performed after ICH and once daily until the rats were sacrificed. Thus, the times of EA treatment for ICH rats in 6 h, 1 d, 3 d, and 7 d group were once, twice, four times, and eight times, respectively. The animals in Sham EA group were fastened to the frame with Sham EA on therapeutic acupoints plus no penetration plus no electrical stimulation. Treatment in each group was performed once a day until the rats were sacrificed.

### 2.4. Neurological Deficit Scores

Neurological deficit scores were evaluated at 6 h, 1 d, 3 d, and 7 d after ICH by an investigator who was blind to the experiment design according to the five-point scale described previously by Longa et al. [26] as follows: score 0 indicated no neurological deficit; score 1 mild focal neurological deficit (with contralateral forelimb flexion); score 2 moderate focal neurological deficit (circling to the contralateral side); score 3 severe focal neurological deficit (falling to the contralateral side); score 4 no spontaneous activity with a depressed level of consciousness or death. Rats with higher score showed more severity of neurological deficits. Only rats with score of 1 to 3 at 6 h after ICH were considered successful models and used in the current study.

### 2.5. Measurement of BBB Permeability

Evans Blue (EB) content in the brain tissue was used to investigate the effect of EA on BBB permeability. After being anesthetized, the rats were intravenously injected with 2% EB dye at a dose of 4 mL·kg^−1^ via the femoral vein. Ninety minutes later, the rats were perfused with 250 mL of normal saline through the left ventricle and then the brains were removed and dissected. Each hemisphere was weighed, put into 3 mL of methanamide, and then heated in 37°C water for 24 h. Samples were then centrifuged for 20 min at 5000 rpm followed by 10 min at 10,000 rpm. The absorbance of the supernatant was measured at 632 nm wavelength with a spectrophotometer.

### 2.6. *In Situ* Immunofluorescent Detection of Cav-1 in Brains

Frozen coronal sections with the hematoma were used for* in situ* detection of Cav-1 with immunofluorescent chemistry. The sections were treated with buffer containing 0.2% Triton (Sigma) and 50 mM phosphate-buffered saline (PBS) for 5 min, antigen retrieval buffer for 5 min, 10% donkey serum for 1 h, and then polyclonal rabbit Cav-1 (1 : 400; CST) overnight at 4°C. After rinsing with PBS, the slides were incubated with Alexa 488 goat anti-rabbit secondary antibody (1 : 400; Invitrogen), a kind of fluorophore-labeled donkey anti-rabbit IgG (H+L) antibodies, for 1 h at 37°C. The Alexa Fluor® dyes to which these antibodies are conjugated provide for extraordinarily bright antibody conjugates. Sections were mounted with antifade mounting medium (Beyotime). Images were acquired using fluorescent microscope (Nikon) at a constant exposure.

### 2.7. Western Blot Analysis of Cav-1 in Brains

Rats were deeply anesthetized and were transcardially perfused with normal saline. Then brains were quickly removed from the skull and stored at −80°C until next steps. Denatured protein samples from the whole frozen coronal sections were resolved in sodium dodecyl sulfate-polyacrylamide gels and transferred to polyvinylidene fluoride membranes (Invitrogen). After blocking, membranes were incubated overnight at 4°C with polyclonal rabbit Cav-1 (1 : 1000; CST) followed by incubation with the goat anti-rabbit horseradish peroxidase-conjugated secondary antibodies (1 : 5000; boyun biotech). Chemiluminescence was detected using ECL advance western blotting detection reagents under the imaging system (MicroChemi).

### 2.8. *In Situ* Zymography for Measuring MMP-2/9 Activity in Brains

Rats were deeply anesthetized and transcardially perfused with normal saline followed by 4% paraformaldehyde. Brains were then postfixed overnight in 4% paraformaldehyde at 4°C. Tissue was frozen in optimum cutting temperature compound and stored at −80°C until sectioning. Tissue was cut into coronal 6 *μ*m thick sections using a cryotome (Thermo Shandon). Gelatinolytic activities of MMP-2/9 in frozen brain sections were measured with* in situ* zymography using EnzCheck collagenase kit (Invitrogen) following the manufacturer's instructions. Frozen brain sections were incubated with the 1x reaction buffer containing 0.8 mg/mL of FITC-labeled DQ gelatin at 37°C overnight. The gelatin was cleaved by MMP-2/9 and yielded the peptides whose fluorescence intensity was detected as the representatives of gelatinolytic activities of MMP-2/9. Fluorescence intensity was measured with a fluorescence microscope at a constant exposure.

### 2.9. Statistics Analysis

All the data were presented as means ± standard (mean ± SD) deviation. Statistics analysis was performed by SPSS 15.0 statistical software. For multiple group experiments, comparisons were made using one-way analysis of variance (ANOVA) and followed by Dunnett test for comparison of two groups within the multiple groups. The significance level was set at *P* < 0.05.

## 3. Results

### 3.1. EA Improved Neurological Function

**Figure 1:**
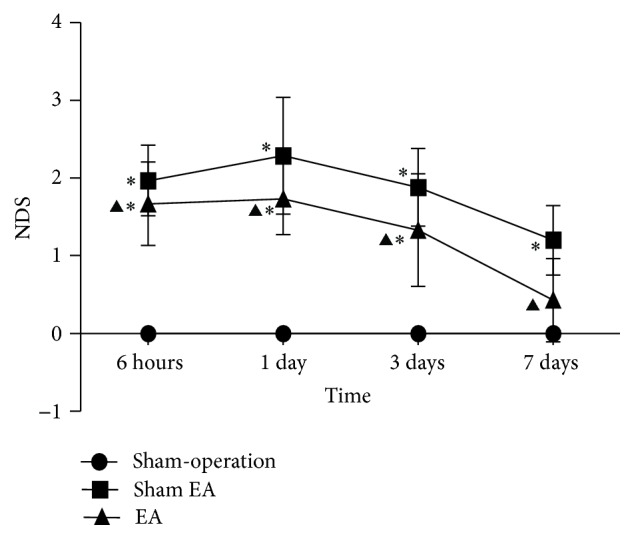
Effects of EA on neurological deficits after ICH in rats (mean ± SD, *n* = 24). NDS in Sham-operation group, Sham EA group, and EA group at 6 h, 24 h, 3 d, and 7 d. One-way analysis of variance (ANOVA) was used for multiple group experiments comparison and followed by Dunnett test for comparison of two groups within the multiple groups. ^*∗*^ *P* < 0.05, compared with Sham-operated group. ^▲^ *P* < 0.05 compared with Sham EA group.

Sham-operated rats did not show visible neurological deficits. Neurological scores increased at 6 h after ICH, peaked at 1 d, and then descended gradually. Compared with Sham-operation group, Sham EA group has significant differences at the time point of 6 h, 1 d, 3 d, and 7 d after ICH (*P* < 0.05). Compared with Sham EA group, EA group has significant differences at 6 h, 1 d, 3 d, and 7 d after ICH (*P* < 0.05). Compared with Sham-operation group, EA group has significant differences at 6 h, 1 d, and 3 d after ICH (*P* < 0.05) (Figure 1).

### 3.2. EA Reduced BBB Disruption

**Figure 2:**
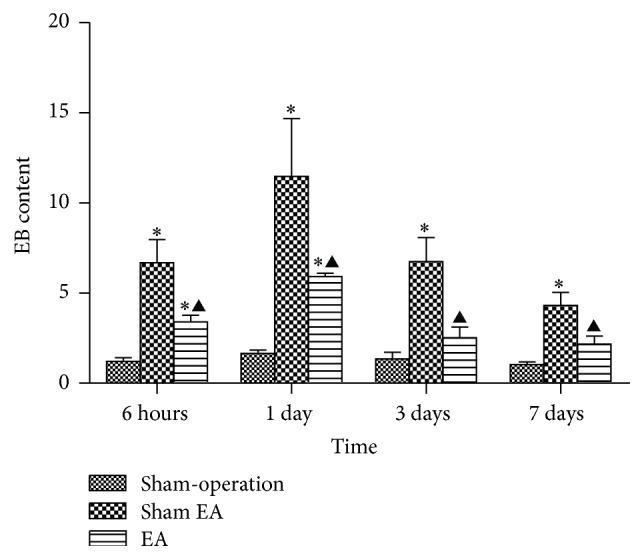
Effects of EA on BBB permeability after ICH in rats (mean ± SD, *n* = 6). Evans Blue leakage experiments for determining BBB permeability after acute ICH in rats in 4 subgroups at 6 h, 24 h, 3 d, and 7 d. One-way analysis of variance (ANOVA) was used for multiple group experiments comparisons and followed by Dunnett test for comparison of two groups within the multiple groups. ^*∗*^ *P* < 0.05, compared with Sham-operated group. ^▲^ *P* < 0.05, compared with Sham EA group.

EB content in the brain increased at 6 h after ICH and peaked at 1 d and then descended gradually but still remained higher than normal at 7 d. Compared with Sham-operation group, Sham EA group has significant differences at the time point of 6 h, 1 d, 3 d, and 7 d after ICH (*P* < 0.05). Compared with Sham EA group, EA group has significant differences at 6 h, 1 d, 3 d, and 7 d after ICH (*P* < 0.05). Compared with Sham-operation group, EA group has significant differences at 6 h and 1 d after ICH (*P* < 0.05) (Figure 2).

### 3.3. EA Downregulated the Expression of Cav-1

**Figure 3:**
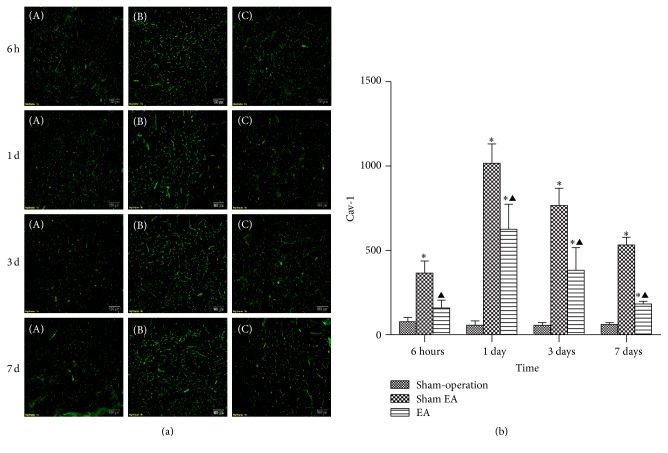
Effects of EA on the* in situ* expression of Cav-1 after ICH in rats (mean ± SD, *n* = 6). (a) Expression of Cav-1 in focal rat brain coronal frozen sections. (A) Sham-operation group; (B) Sham EA group; (C) ICH plus EA group. (b) Quantitative analysis for the results of groups at 6 h, 24 h, 3 d, and 7 d, respectively. One-way analysis of variance (ANOVA) was used for multiple group experiments comparisons and followed by Dunnett test for comparison of two groups within the multiple groups. ^*∗*^ *P* < 0.05, compared with Sham-operated group. ^▲^ *P* < 0.05 compared with Sham EA group.

**Figure 4:**
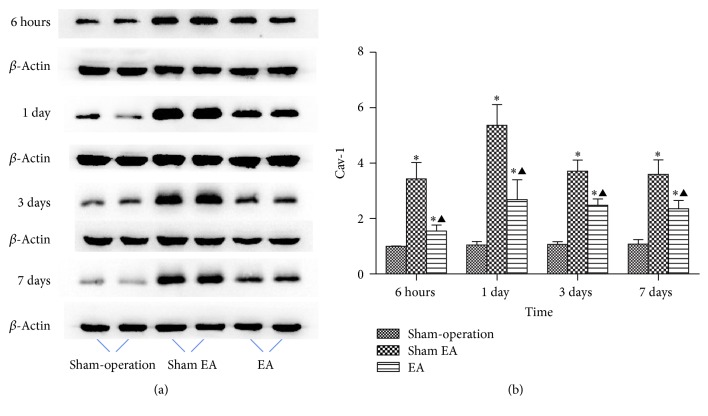
Western blot analysis on the overall expressions of Cav-1 in brain from rats (mean ± SD, *n* = 6). (a) Expression of Cav-1 in brain. (b) Representative statistic results. One-way analysis of variance (ANOVA) was used for multiple group experiments comparisons and followed by Dunnett test for comparison of two groups within the multiple groups. ^*∗*^ *P* < 0.05, compared with Sham-operated group. ^▲^ *P* < 0.05 compared with Sham EA group.

With immunofluorescence and western blot, the results showed that the expression of caveolin-1 increased at 6 h after ICH and peaked at 1 d and then descended gradually but still remained higher than normal at 7 d. Compared with Sham-operation group, Sham EA group has significant differences at the time point of 6 h, 1 d, 3 d, and 7 d after ICH (*P* < 0.05). Compared with Sham EA group, EA group has significant differences at 6 h, 1 d, 3 d, and 7 d after ICH (*P* < 0.05). Compared with Sham-operation group, EA group has significant differences at 6 h, 1 d, 3 d, and 7 d after ICH (*P* < 0.05) (Figures 3 and 4).

### 3.4. EA Decreased the Activity of MMP-2/9

**Figure 5:**
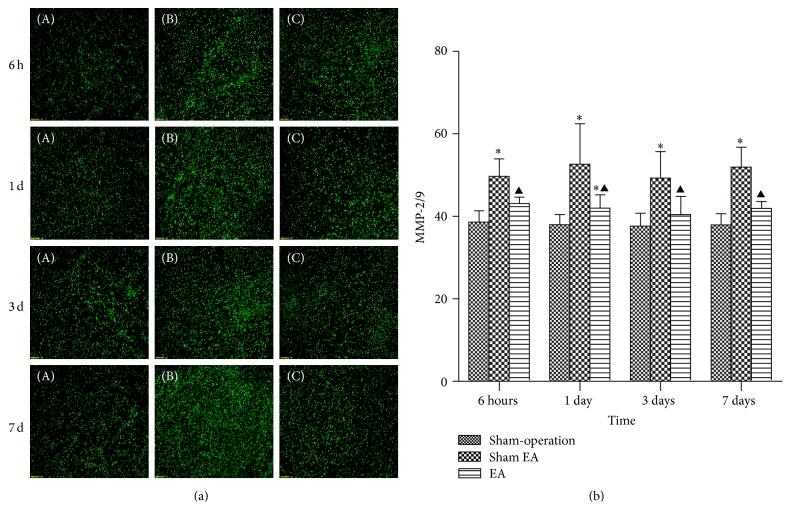
Representative MMPs activity in focal rat brain coronal frozen sections (mean ± SD, *n* = 6). (a) Representative MMPs activity in focal rat brain coronal frozen sections. (A)* In situ* MMPs activity in Sham-operation group; (B) Sham EA group; (C) ICH plus EA group. (b) Quantitative analysis for the results of groups, respectively. One-way analysis of variance (ANOVA) was used for multiple group experiments comparisons and followed by Dunnett test for comparison of two groups within the multiple groups. ^*∗*^ *P* < 0.05, compared with Sham-operated group. ^▲^ *P* < 0.05 compared with Sham EA group.

The MMP-2/9 activity was detected by* in situ* zymography. The result showed that MMP-2/9 activity increased at 6 h after ICH and thereafter remained higher than normal without any trend to decrease or increase. Compared with Sham EA group, EA group and Sham-operation group both have significant differences at the time point of 6 h, 1 d, 3 d, and 7 d after ICH (*P* < 0.05). Compared with Sham-operation group, EA group has differences at 1 d after ICH (*P* < 0.05) (Figure 5).

## 4. Discussion

The present study showed that EA on GV20 could improve neurological function and reduce BBB permeability in acute ICH of rats. This study also provided the mechanism that EA could downregulate the expression of Cav-1 and the activity of MMP-2/9 in a rat model of ICH. These data support the hypothesis that EA exerts neuroprotection via Cav-1/MMPs/BBB permeability pathway in acute ICH.

EA is an extension technique of acupuncture based on traditional acupuncture combined with modern electrotherapy. There are various advantages of EA as its readily quantifiable stimulation parameters of frequency, intensity, and duration [27]. GV20 acupoint belongs to the Du meridian (the government vessel). It locates at the intersection of the line connecting the apexes of the two auricles and the median line of the head, 7 cun directly above the posterior hairline and 5 cun behind the anterior hairline according to the acupuncture theory and the WHO definition [28]. Based on the TCM theory, Baihui is located on the highest place of the head, where all the yang meridians meet [29]. The function of GV20 can tonify yang, lift the spirits, ascend the function of the spleen, eliminate interior wind, clear the mind, and promote resuscitation [30]. Thus, the GV20 is used specifically to treat psychiatric and neurological disorders such as stroke, dizziness, headache, and anxiety. Our previous study revealed that GV20 based acupuncture can substantially reduce neurological deficit and brain edema and may have potential neuroprotective role in animal models of ICH and stroke [11]. Moreover, needling through GV20 towards GB7, as one type of GV20-based scalp penetration needling, is not only one of the most important branches of SA for stroke but also the most commonly used acupuncture method with the advantage of easy positioning [7]. Thus, scalp acupuncture at GV20 towards GB7 is used to treat experimental ICH in the present study.

Preclinical models of ICH are essential for understanding the basic mechanisms of hemorrhage damage and functional recovery thereafter as well as for the initial testing of potential therapies. Up to now, there are three main models commonly used to understand the physiology behind ICH-microballoon insertion, autologous blood injection, and collagenase injection model. Each model has its own advantages and disadvantages. Microballoon insertion can demonstrate the mass effect of a presupposed volume, but it cannot produce such factors as the toxicity of blood elements and cause BBB disruption [31]. Injection of autologous blood with a controlled size of mass can be used for study of mass effect and the effects of blood products. However, the disadvantage is that it fails to mimic the growing hemorrhagic mass, which often occurs in the pathological process of ICH [32]. The collagenase injection model overcomes the above disadvantages and mimics spontaneous ICH more successfully only with disadvantage of diffusing bleeding from small blood vessels rather than bleeding from the arterial source that happens in ICH patients [31]. Thus, we used the collagenase injection model for present study.

Cav-1 plays an important role in brain injury. In our group, we revealed that Cav-1 KO mice with a loss of Cav-1 had remarkably higher BBB permeability than wild-type mice during ischemia-reperfusion injury. In addition, the molecular mechanisms of maintaining BBB integrity in which Cav-1 plays an important role through inhibition of MMPs activity and protection of TJ protein during cerebral ischemia-reperfusion injury have been reported in our previous study [19]. However, up to now there are seldom published data concerning the expression and function of Cav-1 in acute ICH. One study reported that Cav-1 was upregulated at 1 d after ICH in mice [33], whereas another study reported that Cav-1 was downregulated at 1 d but upregulated at 3 d and 7 d after ICH in rats [34]. Therefore, there is no conclusion whether the expression of Cav-1 in acute ICH is increased or decreased. Our experimental results indicate that the expression of Cav-1 began to increase at 6 h after ICH, reached its peak at 1 d, and then decreased, but it remained to be higher than that of Sham-operation group until 7 d. It indicates that ICH resulted in an upregulation of Cav-1 in brain. In addition, compared with Sham EA group, the EA treatment group at all of the observed time points had significant difference, indicating that EA treatment at GV20 can reduce the expression of Cav-1 in acute stage of ICH to exert neuroprotective function in rat model of ICH.

It is known that both of MMP-2 and MMP-9 expression are increased after ICH [35, 36]. In clinical studies, the levels of MMP-2 and MMP-9 were both found to increase after acute ICH, and the increased MMP-9 was associated with perihematomal edema and neurological worsening within the acute stage [37, 38]. The present study indicated that ICH significantly increased the activities of MMP-2/9 at all of the observed time points and EA treatment remarkably inhibited the ICH-induced activities of MMP-2/9 in the brain.

Taken together, the present study demonstrated that EA on GV20 can improve neurological function deficits and reduce BBB permeability after ICH, and the mechanism possibly targets Cav-1/MMP/BBB permeability pathway.
